# Gamification in Physical Education: Evaluation of Impact on Motivation and Academic Performance within Higher Education

**DOI:** 10.3390/ijerph17124465

**Published:** 2020-06-21

**Authors:** Alberto Ferriz-Valero, Ove Østerlie, Salvador García Martínez, Miguel García-Jaén

**Affiliations:** 1 Department of General and Specifics Didactics, University of Alicante, 03690 San Vicente del Raspeig, Spain; salvador.garcia@ua.es; 2 Department of General and Specifics Didactics, Research Group in Physical Education, Fitness and Performance, University of Alicante, 03690 San Vicente del Raspeig, Spain; 3 Research Group DiTePES: Digital Technology in Physical Education and Sports, NTNU-Norwegian University of Science and Technology, NO-7491 Trondheim, Norway; ove.osterlie@ntnu.no; 4 Department of Teacher Education, Faculty of Social and Educational Science, NTNU-Norwegian University of Science and Technology, NO-7491 Trondheim, Norway

**Keywords:** active methodologies, educational innovation, psychosocial factors, motivation, improvement of indicators, academic performance, Classcraft, sport learning

## Abstract

Gamification is an innovative pedagogical approach to addressing problems related to social behaviour, student motivation and academic performance at different educational stages. Therefore, this research aimed to analyse its impact on the motivations and academic performances of university students. The research was carried out in the training of future teachers specialising in physical education during two academic courses. In total, 127 students participated in the study, divided into a gamified experimental group (*n* = 62) and a control group (*n* = 65). The participants completed a questionnaire to assess motivation in physical education before and after the intervention and performed a final exam to assess academic performance. The results indicated an increase in external regulation in the experimental group only. Furthermore, this group achieved significantly better academic performance. The findings of this study suggest that gamified implementation is beneficial for academic performance at the university stage, even though intrinsic motivation does not change. Furthermore, the nature of rewards or punishments, as characteristic of this pedagogical approach, could play an important role in the expected results, since external regulation increased significantly after the intervention.

## 1. Introduction

Gamification has been gaining in popularity within the educational field. Its use as an active methodology has been growing in recent years by creating a novel educational approach to address problems related to social behaviour and student motivation [1,2,3,4,5]. This emerging pedagogical approach is based on the utilisation of the potential of games in order to redesign methodological and didactic elements to increase motivation and adherence to the teaching–learning process, significantly changing the social behaviours of students to ultimately improve their academic performances [6,7,8]. Gamification, an innovative solution inspired by the idea of the use of recreational elements applied in non-recreational contexts [5,9], has been integrated into an educational context from the moment such elements and recreational experiences became part of the design and planning of the educational process [10,11]. These playful elements, that gamify educational contexts, must be tangible for the students (avatars, points, levels, achievements, insignia, etc.), they must create a sense of action and progression (rules, challenges, competition, cooperation, feedback, etc.) and they must regulate the group’s social–emotional competencies (motivations, emotions, obligations, social relationships, etc.) and, consequently, modulate academic performance [6,12].

Within the area of Physical Education (PE), games have traditionally been used, since any motor, popular, traditional or sport game that is proposed in class is configured as the foundation from which to structure the entire teaching–learning process of the curricular contents [13,14,15]. However, the emerging methodological approach of gamification is even more transformative, since it proposes that the class itself, taking advantage of the fact that we are immersed in a digital society, really transforms itself into an active game with digital elements, based on role-playing games and in video games, within which both teachers and students have the opportunity to grow [4,16,17,18,19,20,21,22]. Inquiry into the possible psychological benefits and the effectiveness of the gamification approach within PE is relatively new, scarce and somewhat controversial [11,23]. Previous quantitative and qualitative research among university students show that gamified classes help create a more ideal classroom climate for learning by arousing more interest in and commitment to the subject, so that greater learning, enjoyment and performance are achieved, compared with traditional methodologies that do not use, among other things, new technologies or game-based elements [24,25,26]. However, other studies show a lower degree of motivation and academic performance in other university students when using gamified classes [27,28,29]. In the field of PE in compulsory education, various studies have been carried out that investigate the psychological effects on students using this methodology as a motivational strategy for learning curricular content specific to the area [4,6,30,31]. Some of these studies, using a mixed quantitative–qualitative design with validated and non-validated questionnaires or interviews, showed that gamification fostered students’ enjoyment and motivation, creating meaningful experiences in PE, despite increasing teacher workload [4,30,31]. Other studies, based on experimental or quasi-experimental designs with scales and validated questionnaires showed that gamification impacted positively on students’ dispositional flows and motivations, improving their basic psychological needs and academic performances at school. However, these studies pointed out that gamification is not effective per se, since other game elements could trigger different motivational outcomes [6]. As it can be understood, although the relationship between these psychological and performance aspects has been analysed, the results of these studies are not conclusive. Therefore, more rigorous research is necessary to continue evaluating the real impact of gamification on student motivation and academic performance within the scope of PE.

Motivation is an essential factor for student success. For this reason, self-determination theory (SDT) is frequently used as a macro-theory to understand the role of motivation within the educational environment [32,33,34]. In fact, this theoretical framework has been successfully used in various studies in gamified contexts within PE [4,6,35]. Essentially, this theory recognises three types of regulatory motivations for human behaviour: intrinsic motivation, based on the performance of a behaviour or activity for its inherent satisfaction; extrinsic motivation, referring to the performance of the activity by external recognition or as a means to achieve something; amotivation, that is, the absence of motivation towards the activity [32]. From highest to lowest, self-determination, intrinsic motivation, extrinsic motivation and amotivation are located on a continuum [33]. Thus, intrinsic motivation, recognised as high quality motivation in SDT, is identified with different positive effects within the educational context, such as greater ability to concentrate on the task [36] or higher levels of commitment to the subject [37]. For its part, in relation to extrinsic motivation, different forms of behaviour regulation have been established within it: external regulation, introjected regulation and identified regulation [33]. External regulation includes those behaviours regulated by external incentives, such as rewards or punishments. Introjected regulation is identified with actions associated with the expectations of personal recognition or actions aimed at avoiding certain personal feelings of guilt and anxiety. The identified regulation implies the carrying out of activities voluntarily because the individual considers them important and beneficial for him or her, even if he or she does not enjoy them. Finally, the behaviours based on amotivation refer to actions carried out with no intention of completing the action or with the relative absence of personal motivation towards that behaviour [32]. Gamification has been associated with improved task achievement and higher academic performance by fostering changes in different manifestations of student motivation from a SDT approach [8,38]. In this sense, gamification creates an adequate gamified atmosphere which impacts positively on students’ learning motivations and dispositional flows, thereby supporting their basic psychological needs and ultimately improving their academic performances [6,39,40]. Thus, it could be suggested that this methodological approach might be incorporated into PE classes when looking for an improvement in such psychological and learning aspects in students [41,42]. However, so far there have been few studies that empirically support the effectiveness of gamification in student motivation and performance, so new studies are needed to confirm such claims [43].

Despite the fact that PE is usually a motivating and fun subject for most students, previous studies have shown that quite a few students, especially in the adolescent period, feel amotivation towards the subject, considering it even frustrating or embarrassing because, on many occasions, teachers do not consider the socio–emotional and psychological needs of students in a specific period of pre-adolescent and adolescent development [44,45,46,47]. Based on this, it is reasonable to propose that one of the main challenges for PE teachers would be to provide sufficient motivation for students in their classes, incorporating new methodological approaches to meet the psycho–emotional and social needs of the students. In this sense, gamification is being configured as a key tool to positively influence the different manifestations of motivation, the classroom climate and school performance [4,5,6]. However, it is evident that, currently, empirical research on the use of gamification within the context of PE is still scarce. Additionally, it is essential to propose interventions using these new active methodologies with university students who will be future teachers, in order to determine how it affects these socio–emotional aspects and so that they experience the use of gamification as a pedagogical resource using information and communication technologies (ICT).

For all of the above, the present study was proposed, the primary objective of which was to analyse the impact that the implementation of gamification has on motivation in university students. The second objective was to analyse and compare their academic performances in the subject, evaluating their learning through an objective test of knowledge evaluation. Consequently, it was initially hypothesised that:

**Hypothesise** **(H1).** *The group of students experiencing gamification would increase their intrinsic motivation compared to the control group.*

**Hypothesise** **(H2).** *The group of students experiencing gamification would increase their external regulation compared to the control group.*

**Hypothesise** **(H3).** *The group of students experiencing gamification would decrease their amotivation compared to the control group.*

**Hypothesise** **(H4).** *The experimental group would obtain higher scores in the final exam compared to the control group.*

## 2. Materials and Methods

The research was carried out within the context of physical education teacher education (PETE), in the subject “Physical activity in the natural environment”. This subject falls within the curriculum leading to obtaining the “Degree of Primary Education Teacher”, with reference to the specialty of PE at the University of Alicante (Spain) during two academic courses in 2018 and 2019. The present investigation is based on a quasi-experimental design and with a non-equivalent control group [48]. To contrast the hypotheses presented, two groups of students in the subject were assigned to the control group, CON, and two other groups of students to the experimental group, GAM. The class schedule was 3:00−5:00 p.m. for the first group and 5:00−7:00 p.m. for the second, along the two academic years. The sample was counterbalanced according to the academic course (GAM vs. CON; CON vs. GAM), avoiding possible bias related to the different schedules. The class groups were established by the university itself, following general criteria, such as ratio of students by group and gender.

### 2.1. Sample

Initially 212 university students participated (Table 1). After applying the exclusion criteria, a final sample of 127 students remained (73 boys: 57.5% and 54 girls: 42.5%; average age: 22 ± 3.5 years). These criteria were: (1) regular attendance of class and being evaluated through an ongoing assessment process, that is ≥ 80% of the lessons (CON; *n* = 29, GAM; *n* = 34); (2) not completing the questionnaires pre or post (CON; *n* = 11, GAM; *n* = 8); (3) having an injury (CON; *n* = 2, GAM; *n* = 1). All the participants were informed about the study, approved their participation and agreed to the publishing of the results anonymously after signing a confidentiality agreement.

### 2.2. Instruments

#### 2.2.1. Self-Determined Motivation

**Figure 1:**
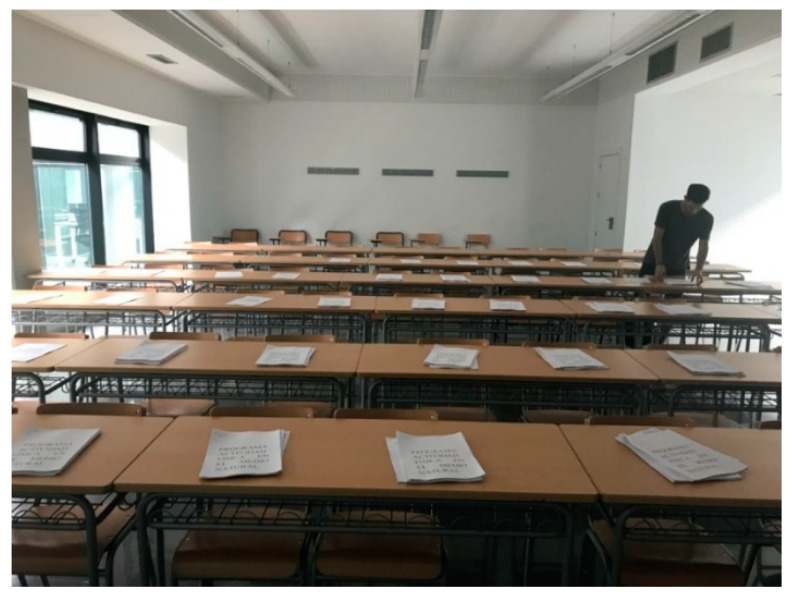
Project explanation and pre-test questionnaire (CMEF-pre).

The questionnaire on Motivation in Physical Education Classes (CMEF) was used to assess motivation [49]. This questionnaire was applied twice: prior to intervention (pre-test) and after intervention (post-test). In the pre-test, the questionnaire was applied before the project explanation, as shown in Figure 1, and therefore before performing the group distribution (GAM and CON), thus avoiding possible biases in the sample. The main statement was adapted to the context of the subject: “I practice physical activity in the natural environment because”. This questionnaire consisted of 20 items, grouped into five factors, which measured intrinsic motivation (e.g., because it is fun), identified regulation (e.g., because I can learn skills that I could use in other areas of my life), introjected regulation (e.g., because it is what I must do to feel good), external regulation (e.g., because it is seen as good by the teacher), and amotivation (e.g., I do not understand why we should do it). These items had a closed response option, following a Likert scale from 1 to 5, with 1 (= totally disagree) to 5 (= totally agree). CMEF was shown to be an excellent fit to the data (CFI = 0.96; TLI = 0.95; GFI = 0.95; SRMR = 0.04; RMSEA = 0.05), and the scale showed satisfactory internal consistency (α > 0.70) [49].

#### 2.2.2. Final Exam

One week after the practical intervention, a theoretical final exam was given to evaluate the academic performance. This exam consisted of a test of 40 multiple choice questions related to the contents studied (identical for both treatment groups), with five possible predetermined answers to choose from. All questions were of equal value. The rating was calculated using the following equation: Rating = Hits × 0.25.

### 2.3. Procedure

**Figure 2:**
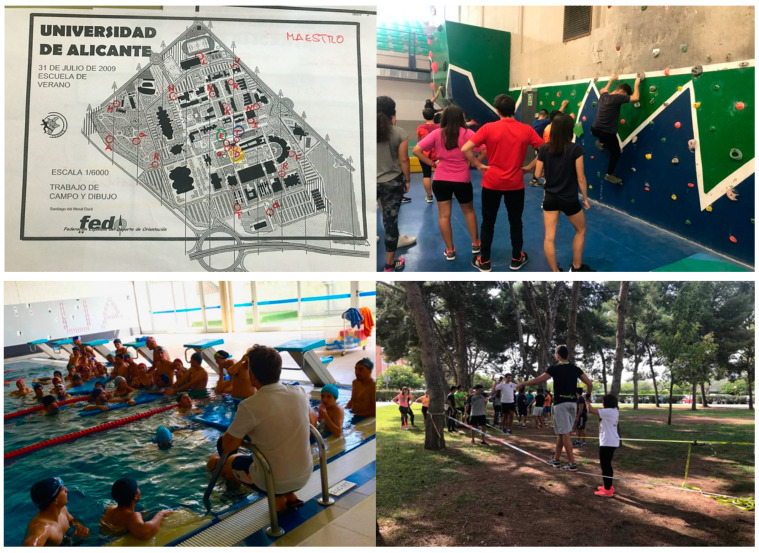
Different lessons carried out during the intervention.

A five-week programme with a total of 30 h was performed, as shown in Table 2. During the first week, the questionnaire (CMEF-pre) was administered to the students and an initial class was held with all the participants to explain the operation of the classes for the following weeks so that, in the case of the experimental group, the students were familiar with the completion of the different questionnaires and the use of the ClassCraft^®^ interface (Classcraft Studios Inc., New York, NY, USA). During weeks two, three and four, the students received different practical classes comprising a total of over 18 h (orienteering, climbing, aquatic skills and natural gymnastics), as shown in Figure 2. Both the GAM and CON groups underwent exactly the same grouping and content learning activities, except for the familiarisation lessons with the gamified resource ClassCraft^®^, which was done as homework for the GAM group. Additionally, the teaching style followed the same criteria for both groups, since the entire intervention programme was carried out by the same teacher (the main author), in order to guarantee that the intervention programme was similarly applied within each group (GAM vs. CON). In the last week (week five), after the practical intervention, the questionnaire (CMEF-post) was administered again. Finally, the participants took the final exam.

For the development of the classes with the experimental group, the Classcraft^®^ educational platform (https://www.classcraft.com) was used. This tool includes a gamified and collaborative learning methodology in the educational proposal. All the participants had their own personal and non-transferable account, which gave them access to create their personalised avatar. With the choice of this avatar, the students could choose between one of three characters: Wizard, Warrior or Healer. Each of these characters had different powers which could help their team or clan. Each team was made up of male and female, and had a maximum of 6 components, which had to include all existing roles. In addition, all the participants had to sign the hero’s pact, a commitment to accepting the rules and decisions of the Grandmaster (the teacher).

Through this gamification tool, it is possible to evaluate certain behaviours or conducts linked to the objectives of the subject. These behaviours define progress through the achievement of points of a different nature: points of experience, health, power, etc., as shown in Table 3.

Each character has strengths and weaknesses and different powers. On the one hand, the healer is suggested or chosen by students who like to help others and can heal by restoring their own health points and those of other members of the team or clan. On the other hand, the warrior has a more offensive character, and therefore is more vulnerable to losing health points more easily. Warriors can take away damage from others and heal themselves. Finally, the magician helps other team members by granting action points and is usually chosen by students who do not usually lose health points easily, since they have less of those points compared to the other characters. Finally, for the development of the classes with the gamified experimental group, the following equivalences shown in the table below were established between behaviours and points, both positive and negative. The equivalences between points and rewards (powers) or punishments (sentences) are shown below in Table 4.

### 2.4. Data Analyses

All continuous variables in the data set were subjected to a normality test (Kolmogorov–Smirnov). The data were further subjected to a chi-square analysis and univariate statistical analysis for non-parametric samples, specifically the Mann–Whitney U test, to assess the differences between the groups (GAM vs. CON) on two occasions: pre and post intervention. Next, the Wilcoxon test was applied to observe the intra-group differences (pre vs. post). The level of significance was established at *p* < 0.05 in all cases. The statistical programmes Statistics Product and Service Solutions (IBM^®^ SPSS^®^ Statistics Version 24.0.0.0) (International Business Machines Corp., Madrid, Spain) and Microsoft Excel^®^ in its 2016 version (Microsoft Corp., Redmond, WD, USA) were used.

## 3. Results

### 3.1. Sample Equivalence

Firstly, a Mann–Whitney U test found no significant differences between the groups in terms of age (Z = −0.200; *p* = 0.841) and attendance (Z = −0.354; *p* = 0.723). A chi-square analysis revealed no differences in the distribution by gender (χ^2^ = 1.706; *p* = 0.191).

### 3.2. Motivation

Before submitting this results section, it was reported that the values represented by the motivational variables assessed were calculated from the sum of the items of the questionnaire (e.g., intrinsic motivation value = item 1 + item 6 + item 11 + item 16), where the minimal result could be 4 and the maximal result could be 20 in each of the variables measured.

**Figure 3:**
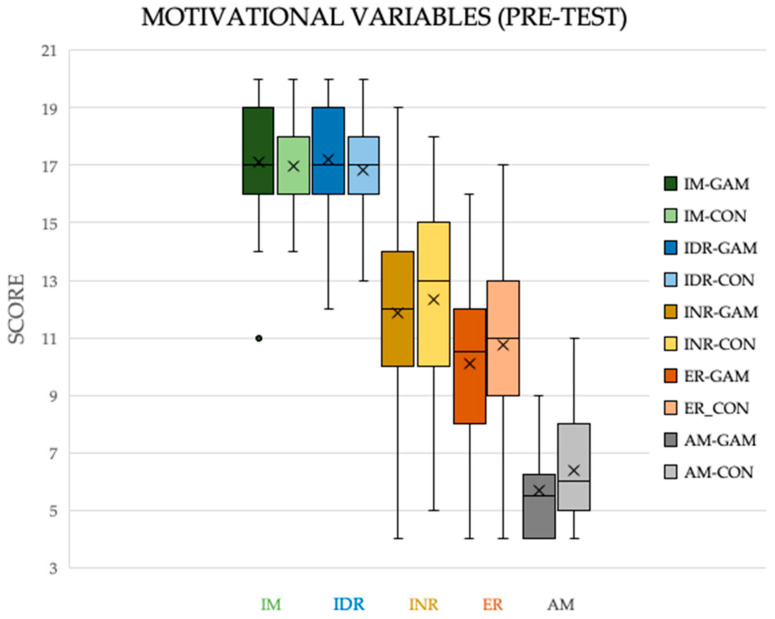
Box diagram of the motivational variables measured in a questionnaire CMEF (pre-test). Note: GAM = experimental group; CON = control group; IM = Intrinsic motivation; IR = Identified Regulation; INR = Introjected Regulation; ER = External regulation; AM = Amotivation.

The different groups did not show initial differences in the motivational variables evaluated, thus, initially the groups were considered homogeneous, as shown in Figure 3.

On the one hand, no significant differences were observed, as shown in Table 5, regarding pre-test and post-test comparisons (*p* < 0.05) in either of the two educational groups, GAM vs. CON, except for the variable of external regulation (ER) in the GAM group, whose post-test value was higher—that is, the experimental group increased its ER (Z = −2.667; *p* = 0.008. ES = 0.34).

On the other hand, although the sample distribution was not parametric, Table 6 shows the means ± SD of all the motivational variables, including minimum and maximum scores pre- and post-test.

**Figure 4:**
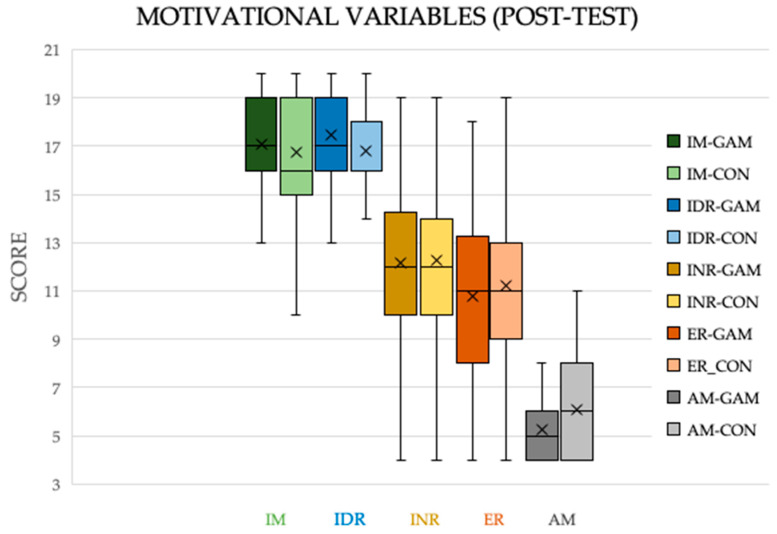
Box diagram of the motivational variables measured in a questionnaire CMEF (post-test). Note: GAM = experimental group; CON = control group; IM = Intrinsic motivation; IR = Identified Regulation; INR = Introjected Regulation; ER = External regulation; AM = Amotivation.

Lastly, a Mann–Whitney U test test showed no post-test differences between the groups (GAM vs. CON) in the motivational variables evaluated after treatment (*p* > 0.05, Figure 4).

### 3.3. Academic Performance

**Figure 5:**
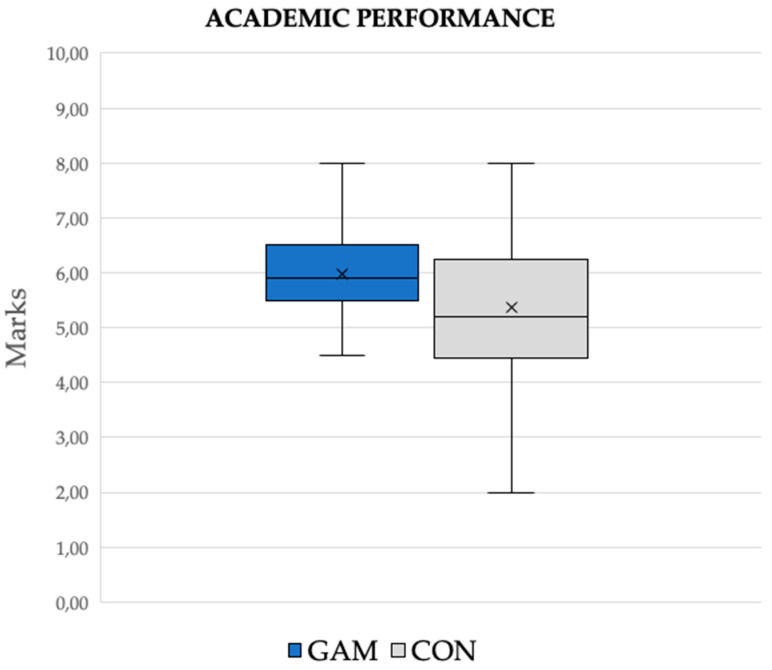
Box diagram of the qualifications obtained in the final exam. Note: GAM = experimental group; CON = control group.

The Mann–Whitney U test showed that the GAM group obtained statistically better scores than the CON group in the results shown in the final exam results (Z = −3417; *p* = 0.001; ES = 0.30), as shown in Figure 5).

A deeper analysis of the data was performed for a comparison of the percentile of the qualifications obtained by both treatment groups, as shown in Table 7. The GAM group obtained a higher rating in all ranks except for the highest percentiles (90 and 95). In addition, these higher qualifications were mostly obtained by males, especially in the CON group, as shown in Table 8. Otherwise, the lowest scores in the GAM group were all observed among females.

## 4. Discussion

Carrying out an intervention based on gamification is based on the idea that any student who participates in this type of proposal can more effectively develop a degree of intrinsic and extrinsic motivation or, conversely, of amotivation [50]. This will have a series of effects on an individual’s psychological attitude and behaviour towards a subject and towards their own learning process, ultimately influencing their academic results [51], among other things. In line with this, Kiesler et al. [17] highlighted gamification as a process that combines two relevant problems of education related to academic results, such as motivation and commitment to the learning process. According to these authors, gamification can not only improve the learning process through better results but will also support three fundamental areas in teaching—emotional, social and cognitive areas [17].

According to the first objective of the research, the obtained results in the questionnaire on Motivation in Physical Education Classes (CMEF) [49] showed a significant increase in the values of extrinsic motivation, specifically ER, only in the group that underwent gamification. This allows us to accept the proposed research hypothesis (H2). As previous research has shown, ER is understood as a powerful form of motivation, although it is difficult to maintain since it describes behaviours regulated by contingencies external to the subject [32,33]. Good and desirable behaviours, as well as undesirable behaviours, by the students in the experimental group were regulated by contingencies external to the subject (rewards and punishments) through the dynamics of the game itself (with experience points -XP- or health points -HP-), detailed in Table 3 and Table 4, while studying the teaching–learning process of the contents of the subject (orienteering, climbing, natural gymnastics, etc.). This feedback, as Bogost [52] points out, must be part of the gamified structure and translate the process that is being developed in the students, so that the students themselves can interpret and control the situation in which they are immersed, activating and improving achievement motivation by receiving information or feedback about its execution. Taking into consideration that university students are already adults and that their education is not compulsory, these rewards or punishments could change their ER as they experience an increase in personal value to those contingencies of the game, as was the case in the group that experienced gamification. A priori, this argumentation could reinforce the opposite idea, that students actively participate in the class for reasons intrinsic to the game, which could be synonymous with enjoyment and, by definition, lead to an increase in intrinsic motivation [33] according to the first hypothesis raised. However, Fernandez-Rio, et al. [4] stated that tangible rewards are a key element for a successful implementation of gamification. Even though the experience points do not represent a functional reward in themselves, the accumulation of these points becomes an academic power, and that could justify the modification of this variable. Unlike similar studies carried out with children and adolescents [42], university life promotes an increase in personal independence and responsibility that could directly relate these academic powers to their qualification [53]. According to this, Hanus and Fox [28] suggested that, in terms of university students, additional rewards are interpreted as controlling, causing students to feel less confident, be less satisfied with the course and have less motivation to engage with the material. Unlike the Hanus and Fox study, the present investigation carried out a gamified intervention from a MDA approach (Mechanic–Dynamic–Aesthetic). However, both rewards and punishment are reflected in the same way—that is, in a public leaderboard (XP, HP and AP) which all students participating in the class can see. This could have a negative effect on social comparisons [54,55]. For these reasons, the features design of the rewards or punishments could become an important attribute for the effective application of gamified treatment. Thus, professionals who apply such interventions must seek to reduce social comparisons and see that the nature of rewards or punishment is inherent in the game itself and not extrapolatable to tangible interests.

Another aspect to highlight in the results of this study, directly related to the first hypothesis, is the absence of change in the IM of the group implemented with gamification. This is somewhat in line with other studies carried out with university students where IM decreased [28]. Despite the fact that gamification was an innovative teaching technique for the students who participated in this study, which could have contributed to improving this variable [56], IM remained without significant change. This allows us to reject the proposed research hypothesis (H1) and accept the null hypothesis (H0).

Despite the fact that the use of external rewards is related to a decrease in intrinsic motivation [34], a recent study in a non-educational context reported that external rewards improved IM as long as the feedback was immediate [57]. During the entire intervention of this research, the researcher used the application Classcraft^®^ for smartphones in order to assign the rewards and punishments immediately through systematic observation, although the students were not aware of this feedback until they started the application at the end of class or when they got home. Therefore, even though the teacher used concurrent feedback for practical purposes, for the student it could be considered delayed or terminal feedback, which would not have the expected effect of the teacher on intrinsic motivation in the gamified group [58]. Other authors, who only assessed this type of intrinsic motivation after gamified treatment [4,28], claimed that the decrease observed in this variable may be due to students who are bored and do not wish to be there, and that rewards and incentives might increase intrinsic motivation. However, for students who are innately interested in the material and already motivated to attend, efforts to gamify the classroom might harm their intrinsic motivation [4,28]. This claim cannot be verified in the present study, nor does it support the fact that no differences have been found between groups at the amotivation levels, whereby we can reject H3. However, the least qualified student observed in the gamified group scored higher than the least qualified student observed in the control group, as shown in Table 7, which seems to support the abovementioned idea that gamification has a greater effect in less interested students. Thus, gamification in PE may be a double-edged sword since, for most students, it is a very interesting and funny curricular subject in itself [4,44].

In line with the previous idea, recent studies that applied gamification in physical education in primary and secondary education [4,42] during a time period greater than the present study (approximately 30 h) reported an improvement in IM through quantitative and qualitative results. According to these results, and those found in the present study, it could be that the age factor, educational level [7,59] or even the level of initial motivation [1,2,4,28] are relevant factors when observing an effective change in IM. However, it is necessary to deepen the knowledge of each of these aspects pertaining to motivation, while increasing investigational requirements, in spite of their complexity, since, in most studies examined, they simply analyse motivation in general.

One of the most applicable results of the present investigation, related to the fourth hypothesis, was that the group that complemented the gamified sessions obtained significantly better scores in the final exam compared to the group that carried out the same sessions without the use of this gamified tool (Z = −3417; *p* = 0.001; ES = 0.30). It also rejects the null hypothesis (H0) and accepts the fourth research hypothesis (H4). Despite the fact that, in the present study there was no change in the IM that could somehow justify this result [1,2,3,44,60,61], other studies with university students who received teaching with gamified techniques also obtained improvements in performance and, therefore, better academic qualifications [20,21,22]. It could create a sense of control and responsibility within students, which brings them closer to the opportunity to replace the usual goal of learning in order to pass with a more simple desire to learn [26]. Accordingly, not only can there be an improvement in academic results or performance, but students can perceive an increase in the retention and acceptance of knowledge [19] and improvements in learning the contents of the subject [18] in following a gamified approach. Nevertheless, it is necessary to delve further into these aspects in future inquiry, especially in PE.

It seems that the effect of a gamified experience does not affect all students equally [29]. When comparing the distribution of academic performances among all of the participants in the different treatment groups, it was observed that the gamified group, on average, was better than the control group in all percentiles, as shown in Table 7, except for the highest ranks (90 and 95 percentile). This suggests that the gamified group achieved higher academic performance in the sample distribution (compared to the control group), although the highest marks were obtained by the control group. In fact, the worst percentile of the gamified group got marks very close to the limit of approval. Accordingly, it is necessary to emphasise that, within these best scores, most were observed among males in both treatment groups, as shown in Table 8. Moreover, also in line with this analysis, the lowest scores in the experimental group were observed among females. Because this study cannot posit any hypothesis on gender differences and no study related to gamification was found regarding a possible different effect on boys and girls in PE, future studies should investigate the effect of gamification and gender interaction in a physical education context.

Some practical implications may be extracted from this study. The use of a gamified teaching approach using a technological resource enables students to participate from an MDA perspective. The configuration of rewards and punishments must be intrinsic and connected to the game itself, avoiding an approach that encourages less autonomous motivation. In addition, the resource used must be in a mature phase and the interface used must be familiar to the students. Finally, meta-analytic evidence suggests that combinations of cooperation and competition are likely to be an effective gamification strategy [50], which can invite us to use points, badges and leader boards (PBL).

## 5. Limitations and Future Lines of Research

The use of Classcraft^®^ as a tool for gamified educational intervention allows both groups (Intervention and Control) to receive exactly the same content with the same methodology (grouping, design of activities, length of classes, types of feedback in class, etc.). At the same time, this becomes a major limitation, since combining an innovative pedagogical approach, such as gamification, and the use of a technological resource to carry it out makes it less possible to discern whether the results are attributed to one (gamification) or the other (technological resource), or both (gamification–technological resource) factors. In addition, in terms of the results where motivation plays a prominent role, the absence of measuring other motivational variables, such as integrated regulation [62,63], is another limitation in which future studies with university students should be considered. Although the present study sample may be sufficient, given the research design, future research could extend the sample by allowing for the analysis of possible divergent effects on boys and girls. Another limitation in the present study is its lack of qualitative data, which could shed more light on the students’ experiences. Future studies in the field of physical education should also investigate the optimal length of intervention as compared to the one applied in the present study.

## 6. Conclusions

As reflected in the literature, it has been observed in the present paper that the pedagogical approach of gamification, implemented in future university teaching PE specialists, shows no variation in intrinsic motivation, but variation in extrinsic motivation is evident. These results are discussed in depth, highlighting the importance of the role that the nature of rewards or punishments could occupy in the implementation of this pedagogical approach to control the change in the extrinsic motivational regulation of students. Gamification is an innovative education approach, which seems to improve learning experiences by improving variables such as motivation. However, this approach seems to have different effects among students, being more effective in less motivated students. Regarding the fourth hypothesis, there is a difference in the scores obtained in academic performance. Therefore, these findings suggest that the implementation of gamification is beneficial for academic performance in university students, even though their intrinsic motivation is not enhanced.

## Figures and Tables

**Table 1:** Descriptive data of the sample (*n*) regarding year, gender and treatment.

Academic Year	Gender	Treatment		Total
		Control	Gamification	
**2018/2019**	Male	11	14	25
	Female	14	17	31
	Total	25	31	56
**2019/2020**	Male	30	18	48
	Female	10	13	23
	Total	40	31	71

**Table 2:** Research design outline.

Weeks	Class	Content	Time (min)	Group	Place
Week 1	1	Motivation questionnaire (pre)	10	Both	Class
		Project explanation	100	Both	Class
	HW	ClassCraft^®^ familiarisation 1	120	GAM Only	Class
	2	Introduction to subject content	110	Both	Class
Week 2	3	Orienteering (Introduction to symbology)	75	Both	Campus
	4	Orienteering (micro-sprint)	75	Both	Campus
	5	Climbing (clothes and material)	75	Both	Climbing wall
	6	Orienteering (relay)	75	Both	Campus
	7	Aquatic Skills (familiarisation and individual activities with flotation material)	50	Both	Swimming pool
Week 3	8	Adventure raid (adapted activities)	75	Both	Campus
	9	Orienteering (score)	75	Both	Campus
	10	Climbing (Boulder)	75	Both	Climbing wall
	11	Orienteering (normal race)	75	Both	Campus
	12	Aquatic skills (individual and group activities with and without flotation material)	50	Both	Swimming pool
Week 4	13	Orienteering (drawing maps)	75	Both	Campus
	14	Natural gymnastics (moving on four legs and jumping)	60	Both	Campus
	15	Natural gymnastics (balance, flexibility and turns)	60	Both	Campus
	16	Climbing (knots and rapel)	75	Both	Climbing wall
	17	Natural gymnastics (complex game without material)	75	Both	Campus
Week 5	18	Motivation questionnaire (post)	10	Both	Class
		Final exam	110	Both	Class
	19	Presentation of results and final discussion	110	Both	Class

Note: HW = Homework.

**Table 3:** Equivalences between behaviours and gamified points.

**Behaviours**	**(+) Points of Experience**		**(-) Health Points**	
	+135 Xp	Class attendance	−10 Hp	Arriving to class late and/or wearing inappropriate clothing
	+270 Xp	Giving constructive criticism to improve work sessions	−20 Hp	Any disruptive behaviour that prevents the class from developing normally
	+540 Xp	Actively collaborating in group work	−30 hp	Lack of respect for teachers, classmates or materials.
	+1080 Xp	Internalising desirable behaviours for other colleagues		

**Table 4:** Equivalences for rewards and punishments and gamified points.

**Rewards/Punishments**	**(+) Academic Powers**		(-) Judgments *
	1080 Xp + 30 Ap	Five extra minutes to finish the theoretical exam of the subject	Five minutes less to finish the theoretical exam on the subject
	1080 Xp + 30 Ap	The teacher discards 50% of multiple non-true answers in a test question	Editing a short video of the subject
	1080 Xp + 30 Ap	The teacher points to the correct answer in a test question	Delivery of a didactic unit before December
			Delivery of a module in a school before the month of December

Note: * The sentences are applied when the students run out of HP points.

**Table 5:** Results for each educational group in the Wilcoxon test (pre-test vs. post-test).

Treatment Groups	Wilcoxon Test	IM	IDR	INR	ER	AM
**GAM** **(*n* = 62)**	Negative ranges	19	17	24	15	19
	Positive ranges	19	21	29	33	13
	Draws	24	24	9	14	30
	Z	−0.05	−1.17	−1.12	−2.67	−1.22
	Asymptotic sig. (bilateral)	0.96	0.24	0.26	0.01	0.22
	Effect size				0.34	
**CON** **(*n* = 65)**	Negative ranges	24	21	30	21	28
	Positive ranges	18	23	23	27	16
	Draws	23	21	12	17	21
	Z	−0.52	−0.22	−0.7	−1.3	−1.24
	Asymptotic sig. (bilateral)	0.61	0.83	0.48	0.19	0.22

Note: IM = Intrinsic motivation; IR = Identified Regulation; INR = Introjected Regulation; ER = External regulation; AM = Amotivation.

**Table 6:** Mean ± standard deviation for each educational group in motivational variables.

TG	V	IM	IDR	INR	ER	AM
**GAM**	Pre	17.11 ± 1.9	17.21 ± 1.9	11.87 ± 2.7	10.10 ± 3.4	5.47 ± 1.7
	Min	11	12	4	4	4
	Max	20	20	19	16	9
	Post	17.06 ± 1.9	17.48 ± 1.9	12.18 ± 3.2	10.81 ± 3.4	5.23 ± 1.5
	Min	13	13	4	4	4
	Max	20	20	19	18	8
**CON**	Pre	16.95 ± 1.7	16.83 ± 1.6	12.34 ± 3.1	10.74 ± 3.1	6.38 ± 1.8
	Min	14	13	5	4	4
	Max	20	20	18	17	11
	Post	16.75 ± 2.3	16.80 ± 1.7	12.28 ± 3.2	11.22 ± 3.3	6.09 ± 1.9
	Min	10	14	4	4	4
	Max	20	20	19	19	11

Note: TG = Treatment groups; V = Variable; IM = Intrinsic motivation; IR = Identified Regulation; INR = Introjected Regulation; ER = External regulation; AM = Amotivation; Min = Minimum score; Max = Maximum score.

**Table 7:** Percentiles of qualifications in final exam (GAM vs. CON).

Treatment Group	Percentile						
	5	10	25	50	75	90	95
**GAM**	4.83	5.00	5.50	5.90	6.50	7.00	7.17
**CON**	3.12	4.00	4.45	5.20	6.25	7.12	7.91

Note: GAM = experimental group; CON = control group.

**Table 8:** Score and gender of best and worst marks in final exam.

Rank		GAM		CON	
		Gender	Score	Gender	Score
**Higher**	1	Female	8.00	Male	8.00
	2	Male	7.30	Male	8.00
	3	Male	7.20	Male	8.00
	4	Male	7.00	Male	7.70
	5	Male	7.00	Male	7.50
**Lower**	1	Female	4.50	Male	2.00
	2	Female	4.70	Female	3.00
	3	Female	4.80	Male	3.00
	4	Female	5.00	Female	3.40
	5	Female	5.00	Male	3.80

Note: GAM = experimental group; CON = control group.
